# Neoadjuvant chemotherapy-induced decrease of prognostic nutrition index predicts poor prognosis in patients with breast cancer

**DOI:** 10.1186/s12885-020-6647-4

**Published:** 2020-02-27

**Authors:** Takaaki Oba, Kazuma Maeno, Daiya Takekoshi, Mayu Ono, Tokiko Ito, Toshiharu Kanai, Ken-ichi Ito

**Affiliations:** Division of Breast and Endocrine Surgery, Department of Surgery, Shinshu University School of Medicine, 3-1-1 Asahi, Matsumoto, Nagano Japan

**Keywords:** Prognostic nutritional index, Disease-free survival, Neoadjuvant chemotherapy, Breast cancer

## Abstract

**Background:**

The prognostic nutritional index (PNI), which is an easily calculated nutritional index, is significantly associated with patient outcomes in various solid malignancies. This study aimed to evaluate the prognostic impact of PNI changes in patients with breast cancer undergoing neoadjuvant chemotherapy (NAC).

**Methods:**

We reviewed patients with breast cancer who underwent NAC and a subsequent surgery for breast cancer between 2005 and 2016. PNI before and after NAC were calculated using the following formula: 10 × serum albumin (g/dl) + 0.005 × total lymphocyte count/mm^3^. The relationship between PNI and prognosis was retrospectively analyzed.

**Results:**

In total, 191 patients were evaluated. There was no significant difference in disease-free survival (DFS) between the pre-NAC PNI high group and the pre-NAC PNI low group (cutoff: 53.1). However, PNI decreased in 181 patients (94.7%) after NAC and the mean PNI also significantly decreased after NAC from 52.6 ± 3.8 pre-NAC to 46.5 ± 4.4 post-NAC (*p* < 0.01). The mean ΔPNI, which was calculated as pre-NAC PNI minus post-NAC PNI, was 5.4. The high ΔPNI group showed significantly poorer DFS than the low ΔPNI group (cut off: 5.26) (*p* = 0.015). Moreover, high ΔPNI was an independent risk factor of DFS on multivariate analysis (*p* = 0.042).

**Conclusions:**

High decrease of PNI during NAC predicts poor prognosis. Thus, maintaining the nutritional status during NAC may result in better treatment outcomes in patients with breast cancer.

## Background

Despite recent improvements in early detection and progress in surgical techniques, chemotherapy, molecular targeting therapy, and endocrine therapy, breast cancer remains the leading cause of cancer death for women [1]. That is why some patients with breast cancer still develop recurrence even after curative resection and neoadjuvant/adjuvant therapy. Therefore, prevention of recurrence and accurate prediction of prognosis are needed to improve patient survival and fully inform patients.

Accumulating evidence suggests that nutritional status has a strong impact on the outcome of cancer treatment [2]. The prognostic nutritional index (PNI), which is calculated via a simple formula using only serum albumin level and lymphocyte cell count in the peripheral blood, is among the most commonly used parameters to evaluate nutritional status [3]. It has been demonstrated that a preoperative low PNI status is both a risk factor for postoperative complications and a predictive factor for poor prognosis among patients with various malignant tumors including gastric, colorectal, lung, pancreatic, and renal cell cancer undergoing surgery [4–10]. However, only few nutritional studies in the treatment for breast cancer have been conducted [11, 12]. Therefore, the significance of PNI in breast cancer still remains unclear.

Neoadjuvant chemotherapy (NAC) has become widely used for patients with locally advanced breast cancer because it has been shown to significantly elevate the rate of breast-conserving surgery by reducing the tumor volume. Further, the prognosis of the patients who underwent NAC is not inferior to those treated with postoperative chemotherapy [13]. In addition to these clinical benefits, NAC also provides important prognostic information such as pathological complete response (pCR) rate, which has been demonstrated to be a prognostic marker in human epidermal growth factor receptor type 2 (HER2)-positive or triple-negative breast cancer (TNBC) [14]. In this regard, NAC could have potential to present other various prognostic markers as well as pCR and we focused on PNI.

It has been reported that chemotherapy leads to malnutrition due to its gastrointestinal adverse effects including anorexia, nausea, vomiting, stomatitis, and diarrhea [15]. Migita et al. reported that a decrease of PNI during NAC in patients with gastric cancer is associated with a worse long-term outcome [16]. However, to date, there has been no study on the impact of changes in PNI on postoperative prognosis in patients with breast cancer who underwent NAC.

As such, the present study aimed to evaluate the prognostic impact of PNI and other nutritional indices in patients with breast cancer. Towards this goal, we evaluated the changes in PNI and other nutritional factors (e.g., serum albumin level and neutrophil/lymphocyte ratio (NLR)) and body mass index (BMI) during NAC and investigated the association between them and patient outcomes.

## Methods

### Patients and study design

This retrospective, single-center study evaluated patients with breast cancer who underwent NAC and subsequent surgery in Shinshu University Hospital between 2005 and 2016. Patients who could not provide detailed laboratory data and those who could not complete NAC or required a treatment delay of ≥2 weeks due to chemotoxicity were excluded.

### Data collection

Data on clinicopathological characteristics, including age, sex, clinical stage at diagnosis, histological type, histological grade (HG), estrogen receptor (ER), progesterone receptor (PgR), HER2 status, NAC regimens, operation procedure, pathological responses to NAC, and presence of recurrence, were collected from the patients’ medical records. Disease-free survival was defined as the time from surgery to the date of locoregional relapse or distant metastases, whichever occurred first.

PNI, the serum albumin level (Alb) (g/dl), NLR, and BMI were used as nutritional parameters in this study. Pre- and post-NAC blood examination data were also obtained. In addition, both body weight and height were obtained at the same day when blood samples were collected. Pre-NAC nutritional values were collected more than 1 week before the beginning of NAC, while post-NAC values were collected at more than 4 weeks after the last administration of NAC. PNI values were calculated using the following formula: 10 × serum albumin value (g/dl) + 0.005 × total lymphocyte counts in the peripheral blood/mm^3^ [3]. NLR values were as the total neutrophil count divided by the total lymphocyte counts, while BMI as patient’s weight (in kilograms) divided by the square of height (meters) [17, 18]. ΔPNI, ΔAlb, ΔNLR, and ΔBMI were calculated as each value on pre-NAC minus that on post-NAC. The receiver operating characteristic (ROC) curve of each prognostic parameter was analyzed to determine the best cut-off value for disease-free survival.

### NAC regimens and surgical methods

Two different NAC regimens were mainly used: (1) anthracycline-based regimens (AC) including EC (60–75 mg/m^2^ epirubicin and 600 mg/m^2^ cyclophosphamide) or FEC (500 mg/m^2^ fluorouracil, 75–100 mg/m^2^ epirubicin, and 500 mg/m^2^ cyclophosphamide) administered every 3 weeks and (2) taxane regimens including triweekly administered docetaxel (DOC) 75 mg/m^2^ or weekly administered paclitaxel (PTX) 80 mg/m^2^. Most of the patients who underwent four cycles of AC were then administered a further four cycles of DOC or PTX. In HER2-positive patients who received taxane regimens, 6 mg/kg (triweekly) or 2 mg/kg (weekly) trastuzumab was simultaneously administered. Surgery was performed within 4–7 weeks after NAC completion. All patients underwent axillary lymph node dissection. The efficacy of NAC was pathologically examined in the surgical specimens. pCR was defined as no evidence of residual invasive carcinoma in the breast tissue regardless of the axillary lymph node status.

### Adjuvant trastuzumab, endocrine, and radiation therapy after surgery

Following surgery, extensional adjuvant trastuzumab (initially 8 mg/kg, followed by 6 mg/kg) was administered every 3 weeks for 12 months to patients with HER2-positive breast cancer. Whole breast irradiation of 50–60 Gy was performed for the patients who underwent breast-conserving surgery, while chest wall and regional lymph node irradiation of 50–60 Gy was performed for the patients with more than three nodal metastases on the postoperative pathological examinations or preoperative imaging examinations including ultrasonography, magnetic resonance imaging, and ^18^ F-fluorodeoxyglucose positron emission tomography. In addition, postmenopausal patients with positive ER or PgR status were treated with aromatase inhibitors for more than 5 years, whereas premenopausal patients were treated with tamoxifen or tamoxifen with luteinizing hormone-releasing hormone agonist.

### Statistical analysis

Categorical and continuous variables were analyzed using Fisher’s exact test and two-sided tests, respectively. Survival curves were estimated using the Kaplan–Meier method, and significant differences in survival were assessed using the log-rank test. Univariate and multivariate analyses with a Cox proportional hazards model were performed to determine significant factors. All statistical analyses were carried out using StatFlex ver.6 (Artech Co., Ltd., Osaka, Japan), and *p* < 0.05 was considered statistically significant.

## Results

### Clinicopathological characteristics and nutrition parameter of patients

In total, 191 patients with a mean age (± standard deviation) of 51.2 ± 10.4 were evaluated. The patient characteristics are shown in Table 1. With respect to clinical stage at diagnosis, 1 (0.5%), 118 (61.8%), and 72 (37.7%) patients had stage I, II, and III disease, respectively. For the pathological classification, 171 patients (89.5%) had invasive ductal carcinoma; 12 patients (6.3%), invasive lobular carcinoma; and 8 patients (4.2%), other special types. As for intrinsic subtype, 107 cases (56.0%) were luminal (ER+ and/or PgR+/HER2-), 37 cases (19.4%) were luminal HER-2 (ER+ and/or PgR+/HER2+), 24 cases (12.6%) were HER2 enriched (ER- and PgR- / HER2+), and 23 cases (12.0%) were TNBC (ER- and PgR−/HER2-). Eleven patients (5.8%) were treated with AC without taxane; 91 patients (47.6%), AC followed by weekly PTX and/or trastuzumab; and 89 patients (46.6%), AC followed by triweekly DOC and/or trastuzumab. Regarding chemotoxicity, 14 patients (7.3%) required a dose reduction of < 20% during NAC. Mastectomy was performed for 128 patients (67.1%), while breast-conserving surgery was performed for 63 patients (32.9%). pCR was obtained in 37 patients (19.4%). The median follow-up period after surgery was 51 months (range, 1–151 months), and 38 patients (19.9%) developed recurrence.

**Table 1:** Clinicopathologic characteristics in patients

Variables		Total	low ΔPNI	high ΔPNI	
		*n* = 191 (%)	*n* = 91 (%)	*n* = 100 (%)	*p* value
Age(mean ± SD)		51.2 ± 10.4	50.9 ± 9.26	51.5 ± 11.3	0.68
Sex(Male/Female)		0/191	0/91	0/100	
Pre-NAC clinical stage	I	1 (0.5%)	1 (1.1%)	0 (0%)	0.67
	II	118 (61.8%)	52 (57.2%)	66 (66.0%)	
	III	72 (37.7%)	38 (41.7%)	34 (34.0%)	
	IV	0 (0%)	0 (0%)	0 (0%)	
Histological type	IDC	171 (89.5%)	81 (89.0%)	90 (90.0%)	0.89
	ILC	12 (6.3%)	7 (7.7%)	5 (5.0%)	
	Special type	8 (4.2%)	3 (3.3%)	5 (5.0%)	
HG	1	54 (28.3%)	18 (19.9%)	36 (36.0%)	0.15
	2	89 (46.6%)	47 (51.6%)	42 (42.0%)	
	3	23 (12.0%)	14 (15.3%)	9 (9.0%)	
	Unknown	25 (13.1%)	12 (13.2%)	13 (13.0%)	
Subtype	Lunimal	107 (56.0%)	51 (56.0%)	56 (56.0%)	0.79
	Luninal HER2	37 (19.4%)	19 (20.9%)	18 (18.0%)	
	HER2 enriched	24 (12.6%)	12 (13.2%)	12 (12.0%)	
	TNBC	23 (12.0%)	9 (9.9%)	14 (14.0%)	
Regimens of NAC	AC	11 (5.8%)	10 (11.0%)	1 (1.0%)	0.02
	AC → PTX and/or HER	91 (47.6%)	52 (57.2%)	39 (39.0%)	
	AC → DOC and/or HER	89 (46.6%)	29 (31.8%)	60 (60.0%)	
Operation procedures	Bt,Ax	128 (67.1%)	59 (64.8%)	69 (69.0%)	0.65
	Bp,Ax	63 (32.9%)	32 (35.2%)	31 (31.0%)	
Pathological response to NAC	non-pCR	154 (80.6%)	78 (85.7%)	76 (76.0%)	0.10
	pCR	37 (19.4%)	13 (14.3%)	24 (24.0%)	
Pre-NAC PNI		52.6 ± 3.8	50.9 ± 3.7	54.1 ± 3.6	< 0.01
Post-NAC PNI		46.5 ± 4.5	48.8 ± 3.9	44.5 ± 4.0	< 0.01
Recurrence		38 (19.9%)	13 (14.3%)	25 (25.0%)	0.06

*NAC* Neoadjuvant chemotherapy, *HG* Histological grade, *IDC* Invasive ductal carcinoma, *ILC* Invasive lobular carcinoma, *AC* Antracycline, *PTX* paclitaxel, *DOC* docetaxel, *HER* Trastuzumab, *Bt* Mastectomy, *Bp* Partial resection of breast, *Ax* Axillary dissection, *NAC* neoadjuvant chemotherapy, *PNI* prognostic nutritional index

**Fig. 1:**
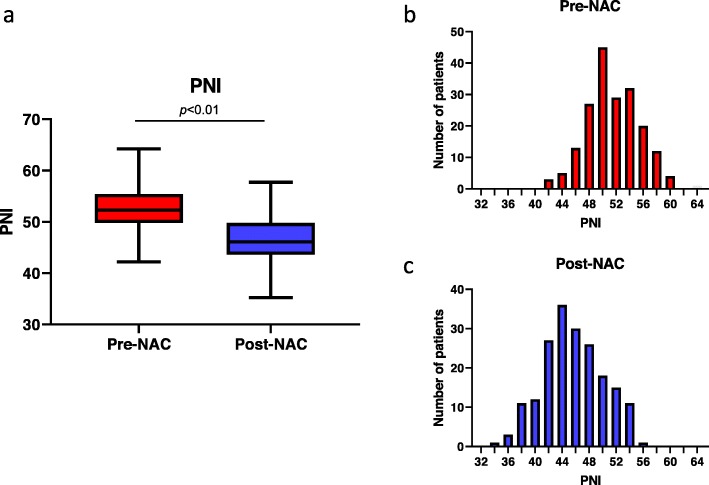
Box-and-whisker plot for Pre-NAC and post-NAC PNI (*p* < 0.01) (**a**). Distribution of pre-NAC (**b**) and post-NAC PNI (**c**). NAC: Neoadjuvant chemotherapy, PNI: Prognostic nutritional index

The mean PNI (pre: 52.6 ± 3.8 vs post: 46.5 ± 4.5; *p* < 0.01) and Alb (pre: 4.41 ± 0.30 vs post: 4.11 ± 0.36; *p* < 0.01) were significantly decreased after NAC, whereas NLR was significantly increased after NAC (pre: 2.50 ± 1.4 vs post: 2.96 ± 1.6; *p* < 0.01). Meanwhile, there was no significant difference in BMI before and after NAC (pre: 22.5 ± 3.9 vs post: 22.3 ± 3.9; *p* = 0.63) (Fig. 1, Additional file 1: Figure S1, Table 2). Among these four factors, PNI was the most commonly decreased (181/191; 94.7%) (Additional file 2: Table S1).

**Table 2:** Comparison of nutritional factors before and after NAC (mean ± standard deviation)

Variables	Pre-NAC	Post-NAC	*p* value
PNI	52.6 ± 3.8	46.5 ± 4.5	< 0.01
Serum albumin level (g/dl)	4.41 ± 0.30	4.11 ± 0.36	< 0.01
NLR	2.50 ± 1.4	2.96 ± 1.6	< 0.01
BMI	22.5 ± 3.9	22.3 ± 3.9	0.63

*NAC* Neoadjuvant chemotherapy, *PNI* Prognostic nutritional index, *NLR* Neutrophil/lymphocyte ratio, *BMI* Body mass index

### Association between nutritional parameters and disease-free survival

**Fig. 2:**
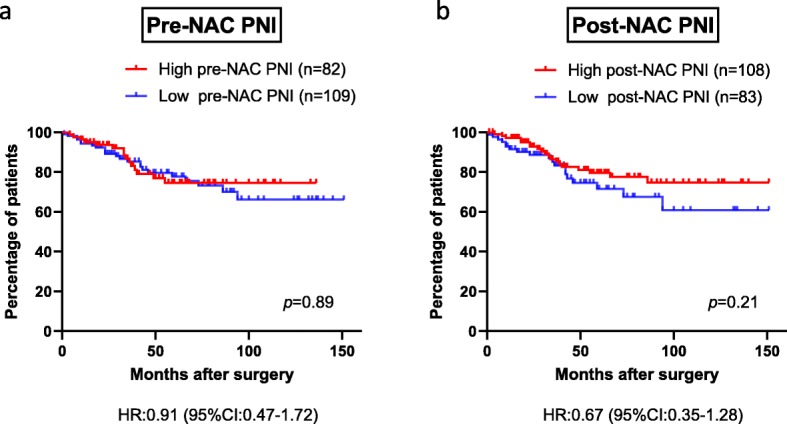
Kaplan–Meier curves for DFS according to PNI at (**a**) pre- (*p* = 0.89) and (**b**) post-NAC (*p* = 0.21). DFS: Disease-free survival, NAC: Neoadjuvant chemotherapy, PNI: Prognostic nutritional index

Disease-free survival in the high and low groups of each nutritional parameter was analyzed to examine the correlation between nutritional status and patient outcome. The optimal cutoff values of PNI, Alb, NLR, and BMI for disease-free survival as identified using the ROC curves were 53.1, 4.36, 2.32, and 21.7 for pre-NAC, respectively, and 45.4, 4.04, 2.57 and 21.5, respectively, for post-NAC (Additional file 3: TableS2). In pre-NAC, there were no significant differences in disease-free survival between the high and low groups for each nutritional parameter (*p* = 0.89 for PNI, *p* = 0.65 for Alb, *p* = 0.25 for NLR, and *p* = 0.76 for BMI) (Fig. 2a, Additional file 4: Figure S2). Similar findings were found on post-NAC (*p* = 0.21 for PNI, *p* = 0.78 for Alb, *p* = 0.58 for NLR, and *p* = 0.58 for BMI) (Fig. 2b, Additional file 5: Figure S3). As well as disease-free survival, disease-specific survival was not different between the high and low groups for each nutritional parameter (Pre-NAC: *p* = 0.21 for PNI, *p* = 0.65 for Alb, *p* = 0.068 for NLR, and *p* = 0.43 for BMI, Post-NAC: *p* = 0.98 for PNI, *p* = 0.14 for Alb, *p* = 0.57 for NLR, and *p* = 0.80 for BMI) (Additional file 6: Figure S4).

### Association between changes of nutrition parameters during NAC and disease-free survival

**Fig. 3:**
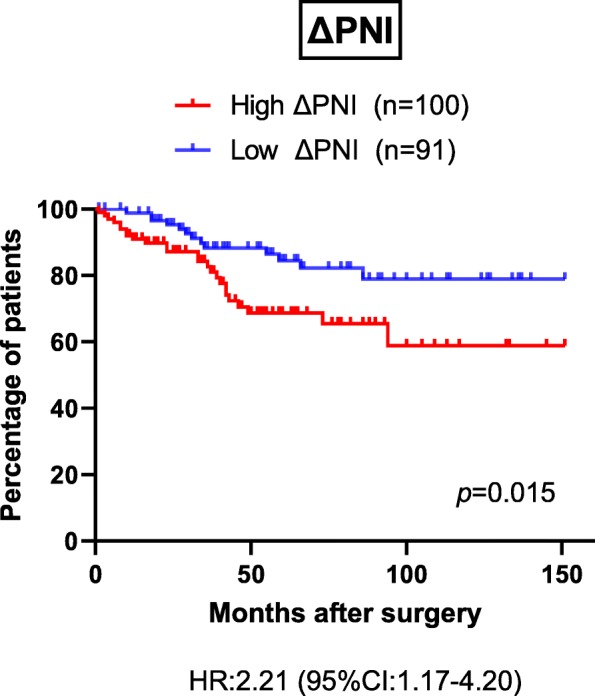
Kaplan–Meier curves for DFS according to change of PNI value (*p* = 0.015). DFS: Disease-free survival, PNI: Prognostic nutritional index.

Next, we focused on the association between changes in nutrition parameters during NAC and disease-free survival. The optimal cutoff value determined via the ROC curve for disease-free survival was 5.26 for ΔPNI, 0.34 for ΔAlb, − 0.17 for ΔNLR, and − 0.26 for ΔBMI (Additional file 3: TableS2). Interestingly, the high ΔPNI group had significantly poorer disease-free survival than the low ΔPNI group (*p* = 0.015) (Fig. 3). Additionally, a trend for lower disease-specific survival was found in the high ΔPNI group than in the low ΔPNI group, although no statistical difference was observed (*p* = 0.14) (Additional file 7: Figure S5). Meanwhile, there were no significant differences in either disease-free survival or disease-specific survival between the high and low groups according to ΔAlb (*p* = 0.053 for disease-free survival, *p* = 0.14 for disease-specific survival), ΔNLR (*p* = 0.65 for disease-free survival, *p* = 0.20 for disease-specific survival), and ΔBMI (*p* = 0.66 for disease-free survival, *p* = 0.66 for disease-specific survival) (Additional file 8: Figure S6, Additional file 9: Figure S7).

The clinicopathological characteristics of the high and low ΔPNI groups are shown in Table 1. The median follow-up period after surgery was 64 (3–151) months for the high ΔPNI group and 43 (1–151) for the low ΔPNI group. The mean age, clinical stage, histological type, HG, subtype, operation procedure, and pathological response to NAC were not significantly different between the two groups. Meanwhile, NAC regimens differed significantly, with a higher rate of patients who underwent NAC with DOC in the high ΔPNI group (*p* = 0.02). Recurrence was more frequent in the high ΔPNI group with marginal significance (*p* = 0.06). In the high ΔPNI group, pre-NAC PNI was significantly higher than that in the low ΔPNI group (*p* < 0.01). Furthermore, the mean ΔPNI was significantly higher in the patients with high pre-NAC PNI than in those with low pre-NAC PNI (Additional file 10: Figure S8), indicating that a large PNI change may be likely to occur in patients with high PNI at baseline.

The higher proportion of patients treated with DOC in the high ΔPNI group prompted us to examine whether NAC regimens affected disease-free survival. However, we found no significant difference in disease-free survival among the three NAC regimens (AC, AC followed by PTX and/or trastuzumab, or AC followed by DOC and/or trastuzumab) (Additional file 11: Figure S9). These data suggest that the difference in disease-free survival by ΔPNI is independent of NAC regimens.

Next, we examined if tumor burden at time of the diagnosis could influence the pre-NAC PNI, post-NAC PNI, or ΔPNI. When we divided the patients into stage I, II and stage III, pre-NAC PNI, post-NAC PNI, or ΔPNI were not different (*p* = 0.87, *p* = 0.73, and *p* = 0.85, respectively), indicating that the volume of disease did not affect either the PNI value or the change in PNI (Additional file 12: Figure S10).

### Association between disease-free survival and ΔPNI based on tumor characteristics

**Fig. 4:**
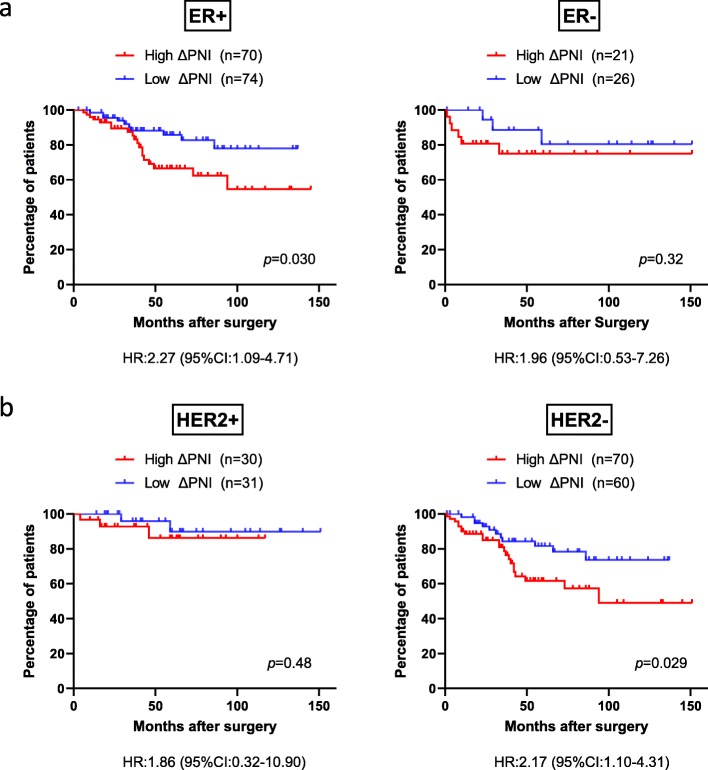
Kaplan-Meier curves for DFS according to the change of PNI distributed by ER and HER2. **a** ER-positive (*p* = 0.030) and negative (*p* = 0.32) breast cancer. **b** HER2-positive (*p* = 0.48) and negative (*p* = 0.029) breast cancer. DFS: Disease-free survival, PNI: Prognostic nutritional index, ER: estrogen receptor, HER2: human epidermal growth factor receptor 2

To investigate whether the effect of ΔPNI on disease-free survival depends on tumor characteristics, we divided the patients according to ER and HER2 expression on tumors. In the ER-positive population, the high ΔPNI group had significantly poorer disease-free survival than the low ΔPNI group (*p* = 0.030) (Fig. 4a). Meanwhile, as for HER2 status, the high ΔPNI group presented significantly poorer disease-free survival than the low ΔPNI group among HER2-negative cases (*p* = 0.029) (Fig. 4b). Disease-free survival was not significantly associated with ER negative (*p* = 0.32) and HER2 positive (*p* = 0.48) status, but the high ΔPNI group tended to have poorer disease-free survival than the low ΔPNI group in both the ER-negative and HER2-positive cohorts (Fig. 4a,b).

On division into four subtypes (luminal; ER+ and/or PgR+ / HER2-, luminal HER2: ER+ and/or PgR+ / HER2+, HER2 enriched: ER- and PgR- / HER2+, and TNBC: ER- and PgR−/HER-), the high ΔPNI group showed a trend of poorer disease-free survival than the low ΔPNI group, although the differences were not significant because of the small number of patients with each subtype (*p* = 0.091 for luminal, *p* = 0.098 for luminal HER2, *p* = 0.67 for HER2 enriched, and *p* = 0.18 for TNBC) (Additional file 13: Figure S11).

Regarding clinical stage, the high ΔPNI group showed significantly poorer disease-free survival than the low ΔPNI group among patients with stage III breast cancer (*p* = 0.0064). In patients with stage I or II breast cancer, the high ΔPNI group tended to show poorer disease-free survival than the low ΔPNI group, although the difference was not significant (*p* = 0.39). As for HG, the high ΔPNI group consistently showed poorer disease-free survival with respect to each HG with marginal or significant differences (*p* = 0.048 for HG1, *p* = 0.072 for HG2, *p* = 0.069 for HG3) (Additional file 14: Figure S12).

### Prognostic factors of disease-free survival

To confirm the significance of ΔPNI in disease-free survival, univariate and multivariate analyses were performed. Univariate analysis revealed that ΔPNI was a significant predictor of disease-free survival (HR: 2.2, 95% CI: 1.14–4.41, *p* = 0.018). Other factors associated with disease-free survival were pre-NAC clinical stage (HR: 3.1, 95% CI: 1.58–5.81, *p* < 0.01) and HER2 status (HR: 0.3, 95% CI: 0.11–0.77, *p* = 0.012). On multivariate analysis using Cox hazard model, ΔPNI was an independent risk factor for disease-free survival (HR: 2.17, 95% CI: 1.08–4.76, *p* = 0.042) (Table 3).

**Table 3:** Univariate and multivariate Cox proportional hazards regression analyses of the clinicopathological parameters

	Univariate			Multivariate		
	*p* value	HR	95% CI	*p* value	HR	95% CI
Age, years (≥50 vs. < 50)	0.73	0.89	0.47–1.68			
Pre-NAC clinical stage (stage I and II vs. stage III)	< 0.01	3.1	1.58–5.81	< 0.01	2.17	1.57–7.27
ER (positive vs. negative)	0.97	1.1	0.48–2.14			
HER2 (positive vs. negative)	0.012	0.3	0.11–0.77	0.016	0.28	0.10–0.79
ΔPNI (high vs. low)	0.018	2.2	1.14–4.41	0.042	2.17	1.08–4.76
ΔAlb (high vs. low)	0.058	1.9	0.97–3.52			
ΔBMI (high vs. low)	0.66	0.87	0.45–1.63			
ΔNLR (high vs. low)	0.66	1.2	0.61–2.18			
Histological type (IDC vs. ILC or special type)	0.23	2.4	0.58–10.03			
Pathological response to NAC (non-pCR vs. pCR)	0.19	0.64	0.32–1.25			

*ER* Estrogen receptor, *HER-2* Human epidermal growth factor receptor type 2, *BMI* Body mass index, *NLR* Neutrophil/lymphocyte ratio, *PNI* Prognostic nutritional index, *IDC* Invasive ductal carcinoma, *ILC* Invasive lobular carcinoma, *NAC* Neoadjuvant chemotherapy, *pCR* Pathological complete response

## Discussion

The present study demonstrates that high ΔPNI is significantly associated with poor disease-free survival and is an independent predictor of disease-free survival. To the best of our knowledge, this is the first report to demonstrate that high ΔPNI is a reliable prognostic factor of disease-free survival in patients with breast cancer who underwent NAC.

Several parameters, including PNI [3], serum albumin level [19], or NLR [20], are used to evaluate nutritional status. Increasing evidence suggests that high preoperative PNI is a predictor of better postoperative complications and patients outcomes in various types of malignancy [4–10]. Meanwhile, both high serum albumin level and low preoperative NLR also have been reported to be associated with better postoperative outcomes in several cancers [19–27]. BMI is also a well-known prognostic factor in breast cancers [28–30], and body weight is also associated with the patients’ nutritional condition [31]. These four factors (i.e., PNI, serum albumin level, NLR, and BMI) are easily calculated or obtained from clinical records or physical examinations. Therefore, we used these four factors as nutritional parameters in the present study.

We found no association between pre-NAC PNI, serum albumin level, NLR, or BMI and disease-free survival. Furthermore, post-NAC PNI, serum albumin level, NLR, or BMI also did not show any correlation with disease-free survival, although the low post-NAC PNI group tended to present poorer disease-free survival than the high post-NAC PNI group. These data indicated that the nutritional index itself did not predict the prognosis either before or after NAC. In general, chemotherapy worsen patients’ nutritional condition due to its adverse gastrointestinal effects [15]. Although some studies have demonstrated significant decreases of various nutritional parameters such as albumin, pre-albumin, and transferrin due to preoperative chemotherapy in cancers of the digestive tract [16, 32], the influence of NAC on the nutritional status of patients with breast cancer has remained unclear. In the present study, we observed significant decreases in PNI and serum albumin level and increases in NLR after NAC. Particularly, PNI was decreased in 181 patients of 191 (94.7%) after NAC. These results suggest that NAC has a negative effect on the nutritional status of patients with breast cancer, and that among the four commonly used parameters, PNI may be the most sensitive parameter to evaluate the nutritional status in patients with breast cancer. Therefore, we consequently focused on changes in the value of these nutritional parameters and found that a decreased PNI after NAC predicts poorer disease-free survival in patients with breast cancer. Significant differences in disease-specific survival were not observed in the present study. However, a trend of poorer disease-specific survival was observed in patients with a high decrease in PNI. A larger-scale study or longer follow-up periods will be able to reveal the differences in disease-specific survival.

In the comparison of clinicopathological characteristics between the high ΔPNI group and the low ΔPNI group, there was a higher percentage of patients who received DOC-containing regimens in the high ΔPNI group than the low ΔPNI group. However, there was no significant difference in disease-free survival among the three NAC regimens (AC only, AC followed by PTX and/or trastuzumab, and AC followed by DOC and/or trastuzumab), indicating that the significant difference in disease-free survival according to ΔPNI does not depend on the type of chemotherapy regimen. One explanation for the higher number of patients who underwent DOC-containing regimens in the high ΔPNI group may simply be due to the stronger gastrointestinal adverse effects of DOC compared with PTX [33, 34]. On the other hand, patients treated with DOC are likely to develop peripheral edema [33, 35], which is associated with hypoalbuminemia. This can be another explanation for the increase of DOC-treated patients in the high ΔPNI group.

The biology of breast cancer is known to depend largely on its intrinsic subtype, which is determined mainly according to ER and HER2 status. Further, it is globally accepted that the prognosis is different between each subtype, and thus the therapeutic strategy depends on the subtype [36]. However, the nutritional status of patients with breast cancer may largely depend on patient factors, and not of the tumor. Consistent with this notion, the present study demonstrated that the influence of ΔPNI on disease-free survival may be similar across all breast cancer subtypes, particularly in patients with ER-positive or HER2-negative breast cancer; however, this should be interpreted cautiously as there was no statistical significance in the number of patients with different subtypes owing to the small number of patients enrolled in this study. Particularly, patients with HER2-positive breast cancer had markedly good disease-free survival to evaluate the statistical difference between those with high and low ΔPNI. This may be due to the administration of trastuzumab that contributed to improved prognosis in patients with HER2-positive breast cancer [37]. As well as intrinsic subtype, clinical stage and HG are also universally accepted as prognostic factors of breast cancer [38, 39]. This study showed that the influence of ΔPNI on disease-free survival is stronger in the advanced stage, although the pre NAC-PNI, post NAC-PNI, and ΔPNI values itself were not dependent on clinical stage. In addition, high ΔPNI is consistently associated with poorer disease-free survival, independent from HG. Although further large-scale studies are required for determining the importance of nutritional change in patient outcomes according to the cancer subtype or the tumor burden, the results of the present study suggest that the association between changes in nutritional status during NAC and patient outcome mainly depends on the patient’s nutritional status, especially in the advanced stage, but not on tumor characteristics.

From the point of view of immunity, better immunological condition has been considered to lead to improved survival in cancer. Malnutrition has been shown to be related to cancer progression due to its association with weak immune response [40, 41]. Accordingly, immune response has also been shown to correlate with better outcomes during various antitumor therapies in breast cancer [42]. Collectively, the result of the present and previous studies supports that maintaining the PNI during NAC may be beneficial to prevent worse prognosis in patients with breast cancer. Several studies have demonstrated that nutritional support such as supplemental immunonutrition containing n-3 polyunsaturated fatty acids enabled improved the nutritional condition of patients who underwent chemotherapy [43–45]. Individual nutritional counseling has also been demonstrated to be important in maintaining the nutritional status [46]. In line with our findings, providing these nutritional support strategies during NAC may result in better patient outcome by maintaining the nutritional condition. Indeed, several clinical trials are ongoing to test whether nutrition interventions could improve the treatment outcome of metastatic breast cancer patients (NCT03045289, NCT03045289). In line with the results of this study, the concept of nutrition intervention should be further broadened to the neo-adjuvant setting.

Several limitations of the present study need to be considered. First, it was a retrospective analysis with a small study population in a single institution. In addition to the heterogeneous nature of breast cancer, the limited number of patients may reduce the statistical power. Second, the NAC regimens varied between patients because the study period spanned several years when treatment regimens changed. Further investigations are therefore needed to validate our results.

## Conclusions

The findings of the present study indicate that a decrease of PNI can be a marker to predict poor prognosis after NAC in patients with breast cancer. Our results imply the importance of monitoring the nutritional status during NAC.

## Supplementary information

**Additional file 1: Figure S1.** Box-and-whisker plot for Alb, NLR, and BMI in pre-NAC and post-NAC. NAC: Neoadjuvant chemotherapy, Alb: Serum albumin level (g/dl), NLR: Neutrophil/lymphocyte ratio, BMI: Body mass index.

**Additional file 2: Table S1.** Distribution of patients with decreased PNI, Alb, and BMI or increased NLR during NAC.

**Additional file 3: Table S2.** The AUC and sensitivity/specificity for ROC curve.

**Additional file 4: Figure S2.** Disease-free survival evaluated using the Kaplan–Meier method for Alb, NLR, and BMI at pre-NAC. NAC: Neoadjuvant chemotherapy, Alb: Serum albumin level (g/dl), NLR: Neutrophil/lymphocyte ratio, BMI: Body mass index.

**Additional file 5: Figure S3.** Disease-free survival evaluated using the Kaplan–Meier method for Alb, NLR, and BMI at post-NAC. NAC: Neoadjuvant chemotherapy, Alb: Serum albumin level (g/dl), NLR: Neutrophil/lymphocyte ratio, BMI: Body mass index.

**Additional file 6: Figure S4.** Disease-specific survival evaluated using the Kaplan–Meier method for Alb, NLR, and BMI at pre-NAC and post-NAC. NAC: Neoadjuvant chemotherapy, PNI: Prognostic nutritional index, Alb: Serum albumin level (g/dl), NLR: Neutrophil/lymphocyte ratio, BMI: Body mass index.

**Additional file 7: Figure S5.** Disease-specific survival evaluated using the Kaplan–Meier method according to change of PNI value. PNI: Prognostic nutritional index.

**Additional file 8: Figure S6.** Kaplan–Meier curves for disease-free survival according to change in Alb, NLR, and BMI. Alb: Serum albumin level (g/dl), NLR: Neutrophil/lymphocyte ratio, BMI: Body mass index.

**Additional file 9: Figure S7.** Kaplan–Meier curves for disease-specific survival according to change in Alb, NLR, and BMI. Alb: Serum albumin level (g/dl), NLR: Neutrophil/lymphocyte ratio, BMI: Body mass index.

**Additional file 10: Figure S8.** Disease-free survival evaluated using the Kaplan–Meier method according to NAC regimens. NAC: Neoadjuvant chemotherapy, AC: Anthracycline, PTX: paclitaxel, DOC: Docetaxel.

**Additional file 11: Figure S9.** Box-and-whisker plot for ΔPNI stratified by pre-NAC PNI. NAC: Neoadjuvant chemotherapy, PNI: Prognostic nutritional index.

**Additional file 12: Figure S10.** Box-and-whisker plot for pre-NAC PNI, post-NAC PNI, and ΔPNI stratified by clinical stage. NAC: Neoadjuvant chemotherapy, PNI: Prognostic nutritional index.

**Additional file 13: Figure S11.** Kaplan–Meier curves for disease-free survival according to change of PNI by breast cancer subtype. PNI: Prognostic nutritional index.

**Additional file 14: Figure S12.** Kaplan–Meier curves for disease-free survival according to change of PNI by clinical stage and HG. PNI: Prognostic nutritional index, HG: Histological grade.

## Data Availability

The data supporting the findings of this work are available from the authors upon reasonable request.

## Abbreviations

AC: Anthracycline
Alb: Serum albumin level
BMI: Body mass index
DOC: Docetaxel
EC: Epirubicin and cyclophosphamide
ER: Estrogen receptor
FEC: Fluorouracil, epirubicin, and cyclophosphamide
HER2: Human epidermal growth factor receptor type 2
HG: Histological grade
NAC: Neoadjuvant chemotherapy
NLR: Neutrophil/lymphocyte ratio
pCR: Pathological complete response
PgR: Progesterone receptor
PNI: Prognostic nutritional index
PTX: Paclitaxel
ROC: Receiver operating characteristics
TNBC: Triple-negative breast cancer
