# Supplementary figures and images for: Neoadjuvant chemotherapy-induced decrease of prognostic nutrition index predicts poor prognosis in patients with breast cancer
Source: BMC Cancer. 2020 Feb 27;20:160. doi: 10.1186/s12885-020-6647-4 (PMC7045374; doi:10.1186/s12885-020-6647-4)

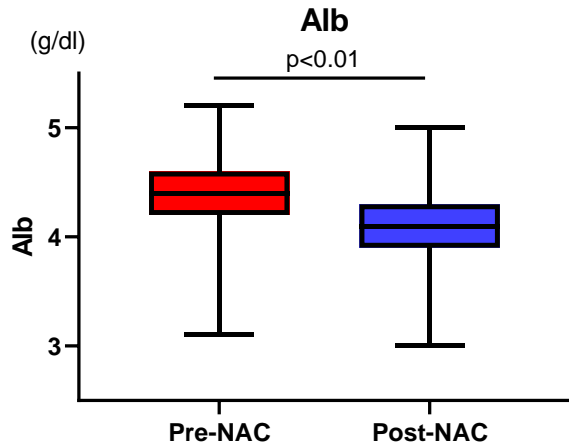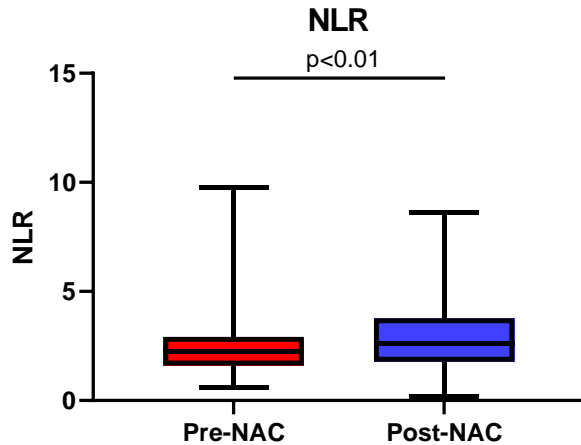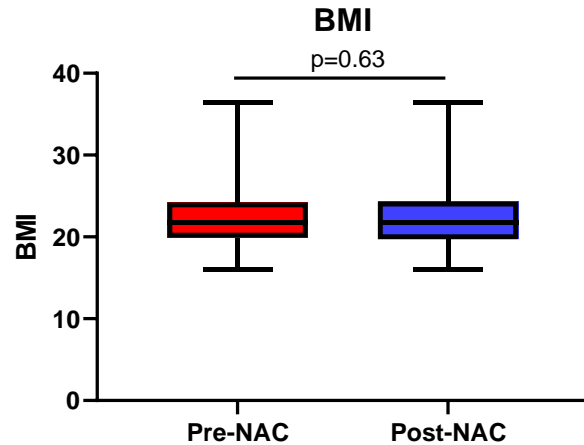

Supplement: Supplementary file 1 — Additional file 1: Figure S1. Box-and-whisker plot for Alb, NLR, and BMI in pre-NAC and post-NAC. NAC: Neoadjuvant chemotherapy, Alb: Serum albumin level (g/dl), NLR: Neutrophil/lymphocyte ratio, BMI: Body mass index. [file 12885_2020_6647_MOESM1_ESM.pdf]

# Disease-specific survival

**$\Delta$ PNI**

High  $\Delta$ PNI (n=100)

Low  $\Delta$ PNI (n=91)

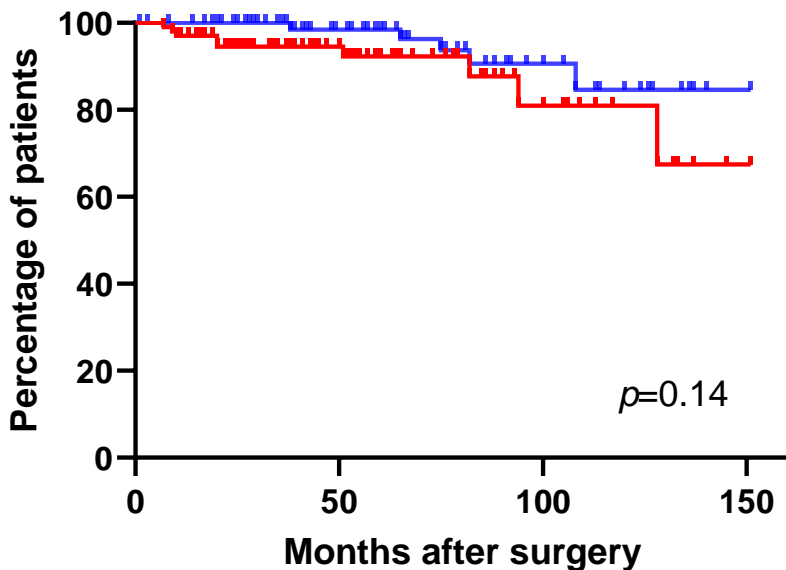

$p=0.14$

HR:2.20 (95%CI:0.77-6.39)

Supplement: Supplementary file 7 — Additional file 7: Figure S5. Disease-specific survival evaluated using the Kaplan–Meier method according to change of PNI value. PNI: Prognostic nutritional index. [file 12885_2020_6647_MOESM7_ESM.pdf]

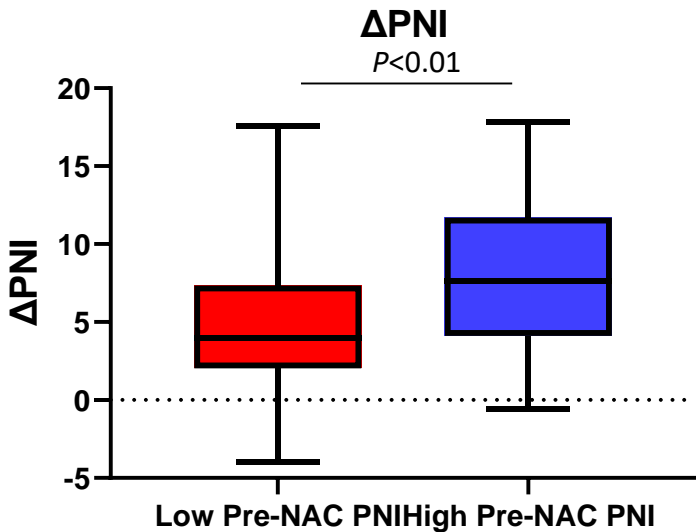

Supplement: Supplementary file 10 — Additional file 10: Figure S8. Disease-free survival evaluated using the Kaplan–Meier method according to NAC regimens. NAC: Neoadjuvant chemotherapy, AC: Anthracycline, PTX: paclitaxel, DOC: Docetaxel. [file 12885_2020_6647_MOESM10_ESM.pdf]

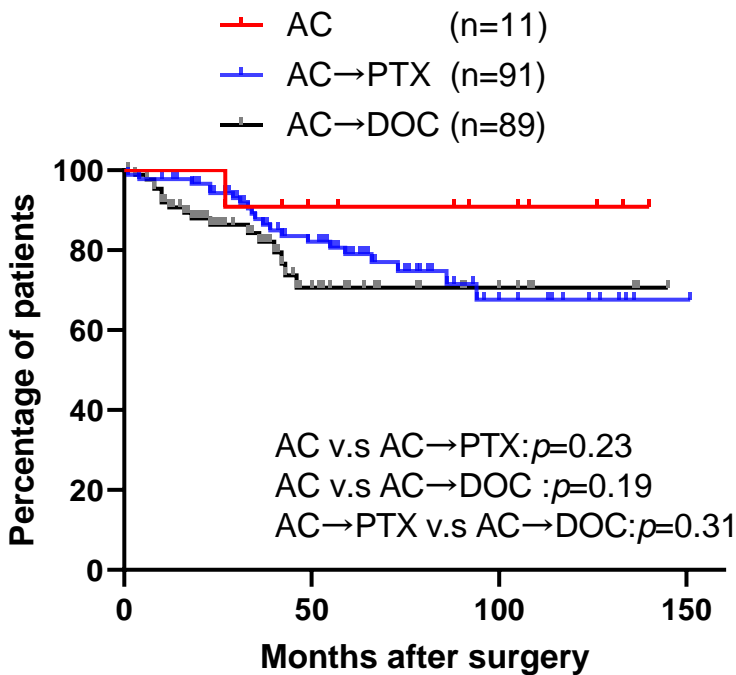

Supplement: Supplementary file 11 — Additional file 11: Figure S9. Box-and-whisker plot for ΔPNI stratified by pre-NAC PNI. NAC: Neoadjuvant chemotherapy, PNI: Prognostic nutritional index. [file 12885_2020_6647_MOESM11_ESM.pdf]

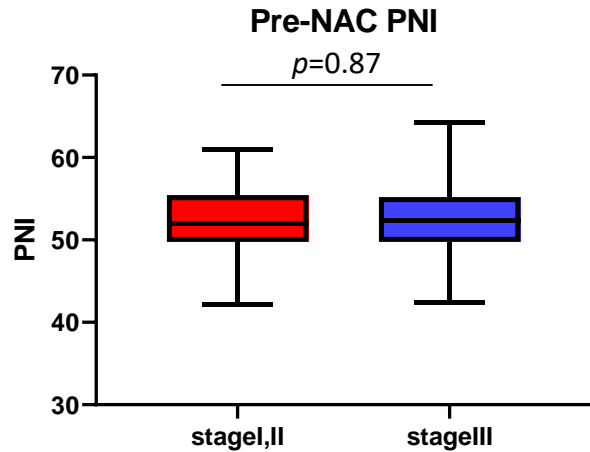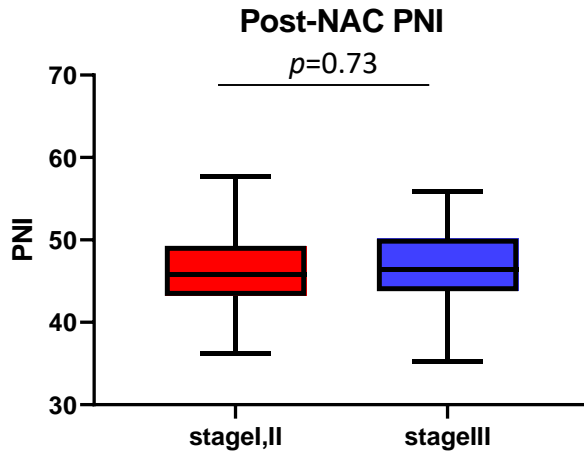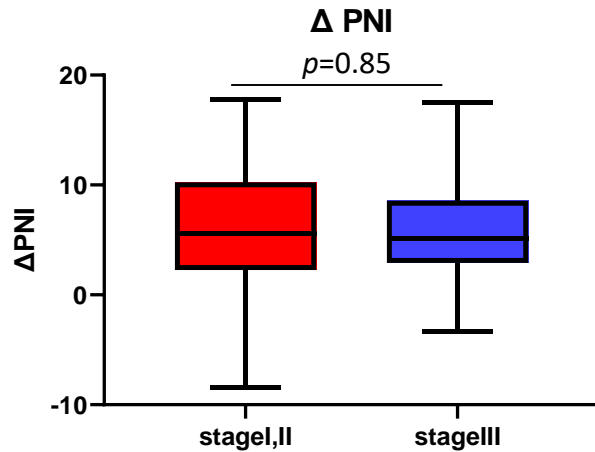

Supplement: Supplementary file 12 — Additional file 12: Figure S10. Box-and-whisker plot for pre-NAC PNI, post-NAC PNI, and ΔPNI stratified by clinical stage. NAC: Neoadjuvant chemotherapy, PNI: Prognostic nutritional index. [file 12885_2020_6647_MOESM12_ESM.pdf]

### Luminal

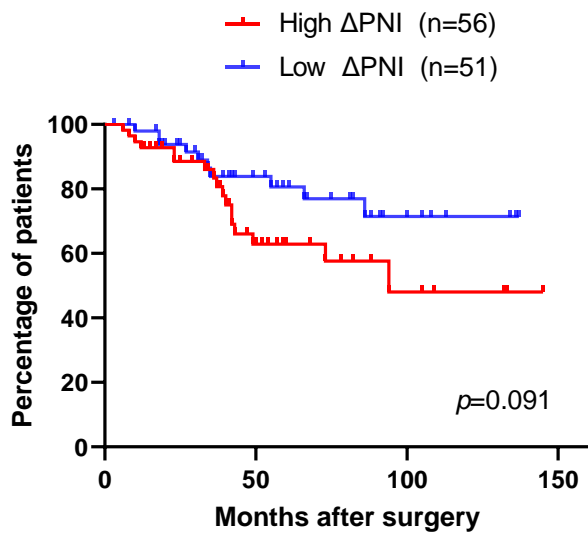

HR:1.98 (95%CI:0.90-4.10)

### Luminal HER2

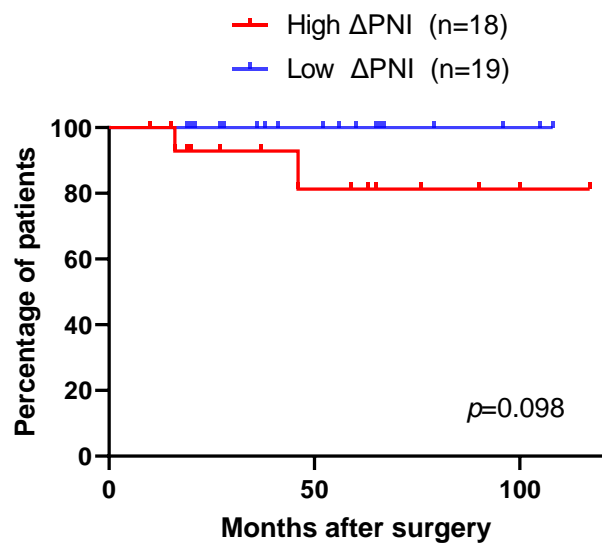

### HER2 enriched

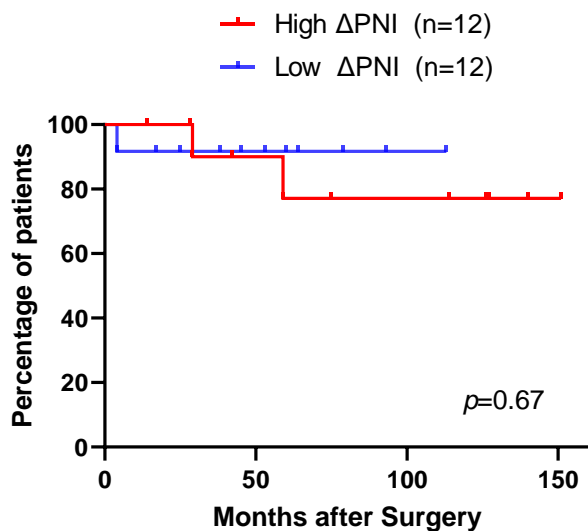

HR:1.66 (95%CI:0.17-16.31)

### TNBC

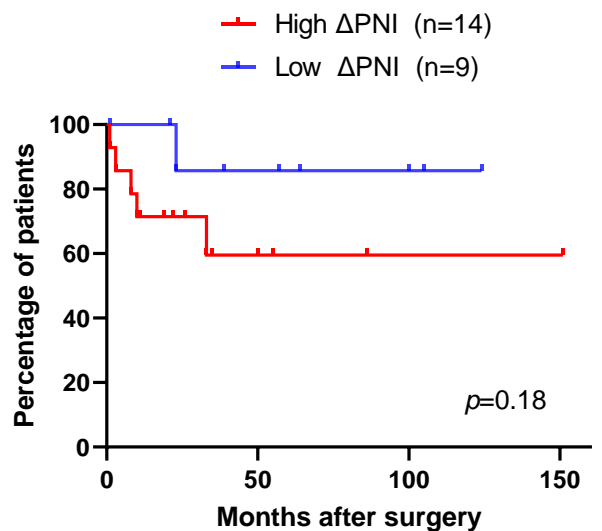

HR:3.80 (95%CI:0.75-19.14)

Supplement: Supplementary file 13 — Additional file 13: Figure S11. Kaplan–Meier curves for disease-free survival according to change of PNI by breast cancer subtype. PNI: Prognostic nutritional index. [file 12885_2020_6647_MOESM13_ESM.pdf]
