# Supplementary material for: Neoadjuvant chemotherapy-induced decrease of prognostic nutrition index predicts poor prognosis in patients with breast cancer
Source: BMC Cancer. 2020 Feb 27;20:160. doi: 10.1186/s12885-020-6647-4 (PMC7045374; doi:10.1186/s12885-020-6647-4)
Supplement: Supplementary file 2 — Additional file 2: Table S1. Distribution of patients with decreased PNI, Alb, and BMI or increased NLR during NAC. [file 12885_2020_6647_MOESM2_ESM.docx]

| Table S1: Distribution of patients with decreased PNI, Alb, and BMI or increased NLR during NAC | | |  |
| --- | --- | --- | --- |
| Variables | N (%) |  |  |
| PNI | 181 (94.7%) |  |  |
| Alb | 140 (72.9%) |  |  |
| NLR | 110 (57.2%) |  |  |
| BMI | 111 (58.2%) |  |  |
| NAC: Neoadjuvant chemotherapy, Alb: Serum albumin level (g/dl), PNI: Prognostic nutritional index, NLR: Neutrophil/lymphocyte ratio, BMI: Body mass index | | | |
