# Supplementary material for: Neoadjuvant chemotherapy-induced decrease of prognostic nutrition index predicts poor prognosis in patients with breast cancer
Source: BMC Cancer. 2020 Feb 27;20:160. doi: 10.1186/s12885-020-6647-4 (PMC7045374; doi:10.1186/s12885-020-6647-4)
Supplement: Supplementary file 3 — Additional file 3: Table S2. The AUC and sensitivity/specificity for ROC curve. [file 12885_2020_6647_MOESM3_ESM.docx]

| Table S2: The area under the curve (AUC) and sensitivity/specificity for receiver operator characteristic (ROC) curve | | | | |
| --- | --- | --- | --- | --- |
| Variables |  | AUC | Sensitivity/specificity |  |
| Pre-NAC | PNI | 0.51 | 0.52 |  |
| Pre-NAC | Alb | 0.52 | 0.52 |  |
| Pre-NAC | NLR | 0.54 | 0.54 |  |
| Pre-NAC | BMI | 0.54 | 0.53 |  |
| Post-NAC | PNI | 0.57 | 0.53 |  |
| Post-NAC | Alb | 0.58 | 0.55 |  |
| Post-NAC | NLR | 0.53 | 0.53 |  |
| Post-NAC | BMI | 0.56 | 0.54 |  |
| Δ PNI | | 0.62 | 0.57 |  |
| Δ NLR | | 0.58 | 0.57 |  |
| Δ NLR | | 0.53 | 0.51 |  |
| Δ BMI | | 0.51 | 0.51 |  |
| NAC: Neoadjuvant chemotherapy, AUC : Area under the curve,  PNI: Prognostic nutritional index, Alb: Serum albumin level (g/dl),  NLR: Neutrophil/lymphocyte ratio, BMI: Body mass index | | | | |
