# Supplementary material for: Neoadjuvant chemotherapy-induced decrease of prognostic nutrition index predicts poor prognosis in patients with breast cancer
Source: BMC Cancer. 2020 Feb 27;20:160. doi: 10.1186/s12885-020-6647-4 (PMC7045374; doi:10.1186/s12885-020-6647-4)
Supplement: Supplementary file 5 — Additional file 5: Figure S3. Disease-free survival evaluated using the Kaplan–Meier method for Alb, NLR, and BMI at post-NAC. NAC: Neoadjuvant chemotherapy, Alb: Serum albumin level (g/dl), NLR: Neutrophil/lymphocyte ratio, BMI: Body mass index. [file 12885_2020_6647_MOESM5_ESM.pdf]

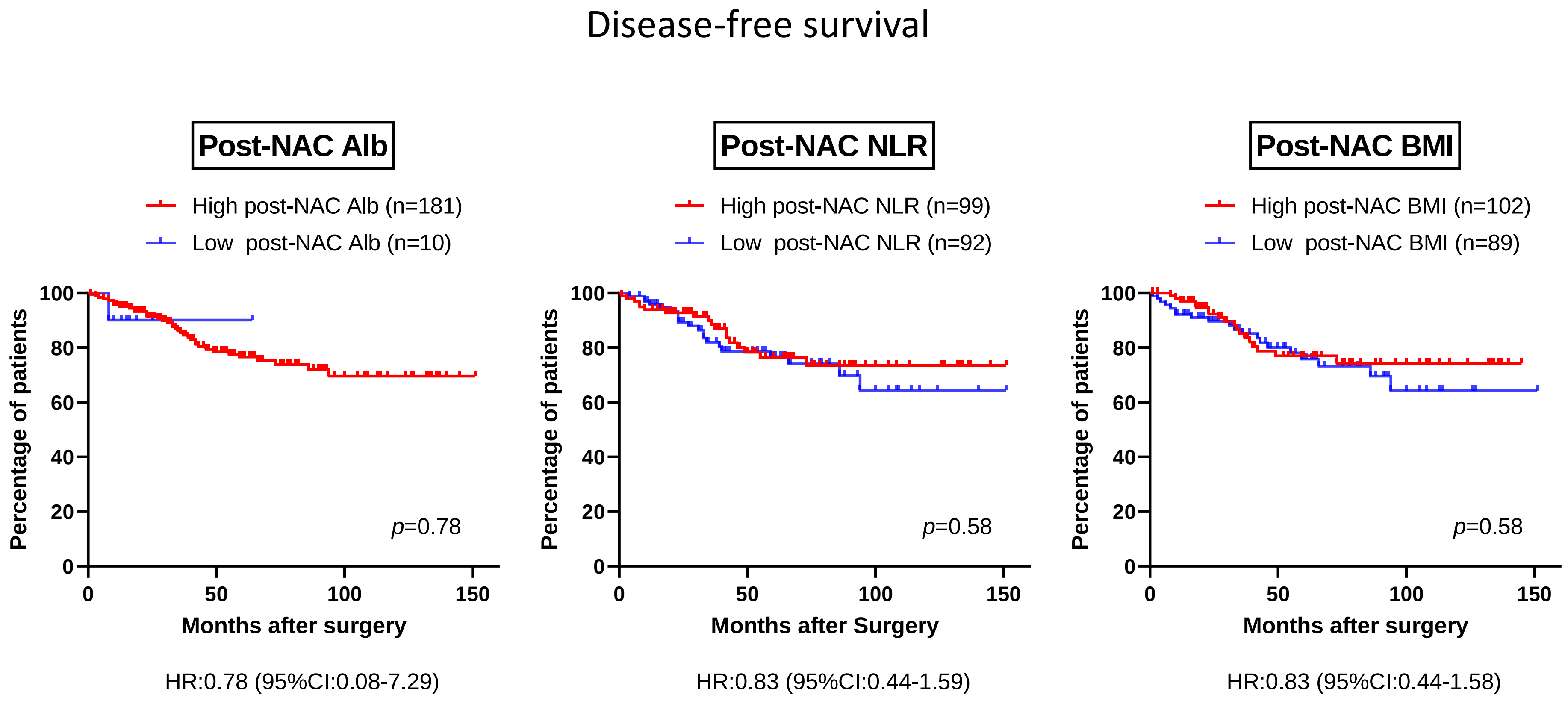
Disease-free survival
Post-NAC Alb
High post-NAC Alb (n=181)
Low post-NAC Alb (n=10)
Percentage of patients
100
80
60
40
20
0
0
50
100
150
Months after surgery
p=0.78
HR:0.78 (95%CI:0.08-7.29)
Post-NAC NLR
High post-NAC NLR (n=99)
Low post-NAC NLR (n=92)
Percentage of patients
Months after Surgery
p=0.58
HR:0.83 (95%CI:0.44-1.59)
Post-NAC BMI
High post-NAC BMI (n=102)
Low post-NAC BMI (n=89)
Percentage of patients
Months after surgery
p=0.58
HR:0.83 (95%CI:0.44-1.58)
