# Supplementary material for: Neoadjuvant chemotherapy-induced decrease of prognostic nutrition index predicts poor prognosis in patients with breast cancer
Source: BMC Cancer. 2020 Feb 27;20:160. doi: 10.1186/s12885-020-6647-4 (PMC7045374; doi:10.1186/s12885-020-6647-4)

Disease-specific survival

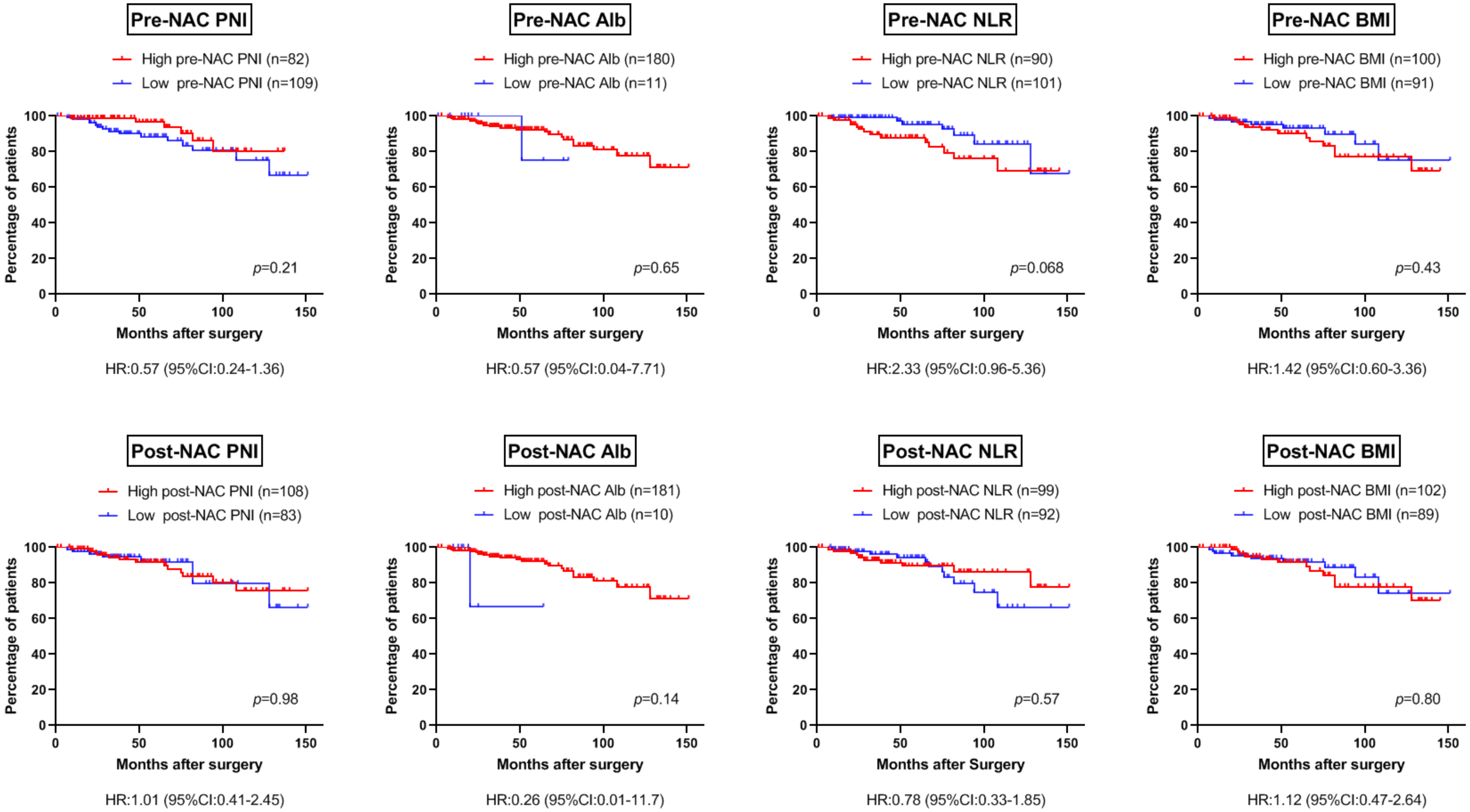

Supplement: Supplementary file 6 — Additional file 6: Figure S4. Disease-specific survival evaluated using the Kaplan–Meier method for Alb, NLR, and BMI at pre-NAC and post-NAC. NAC: Neoadjuvant chemotherapy, PNI: Prognostic nutritional index, Alb: Serum albumin level (g/dl), NLR: Neutrophil/lymphocyte ratio, BMI: Body mass index. [file 12885_2020_6647_MOESM6_ESM.pdf]
