# Supplementary material for: Neoadjuvant chemotherapy-induced decrease of prognostic nutrition index predicts poor prognosis in patients with breast cancer
Source: BMC Cancer. 2020 Feb 27;20:160. doi: 10.1186/s12885-020-6647-4 (PMC7045374; doi:10.1186/s12885-020-6647-4)
Supplement: Supplementary file 8 — Additional file 8: Figure S6. Kaplan–Meier curves for disease-free survival according to change in Alb, NLR, and BMI. Alb: Serum albumin level (g/dl), NLR: Neutrophil/lymphocyte ratio, BMI: Body mass index. [file 12885_2020_6647_MOESM8_ESM.pdf]

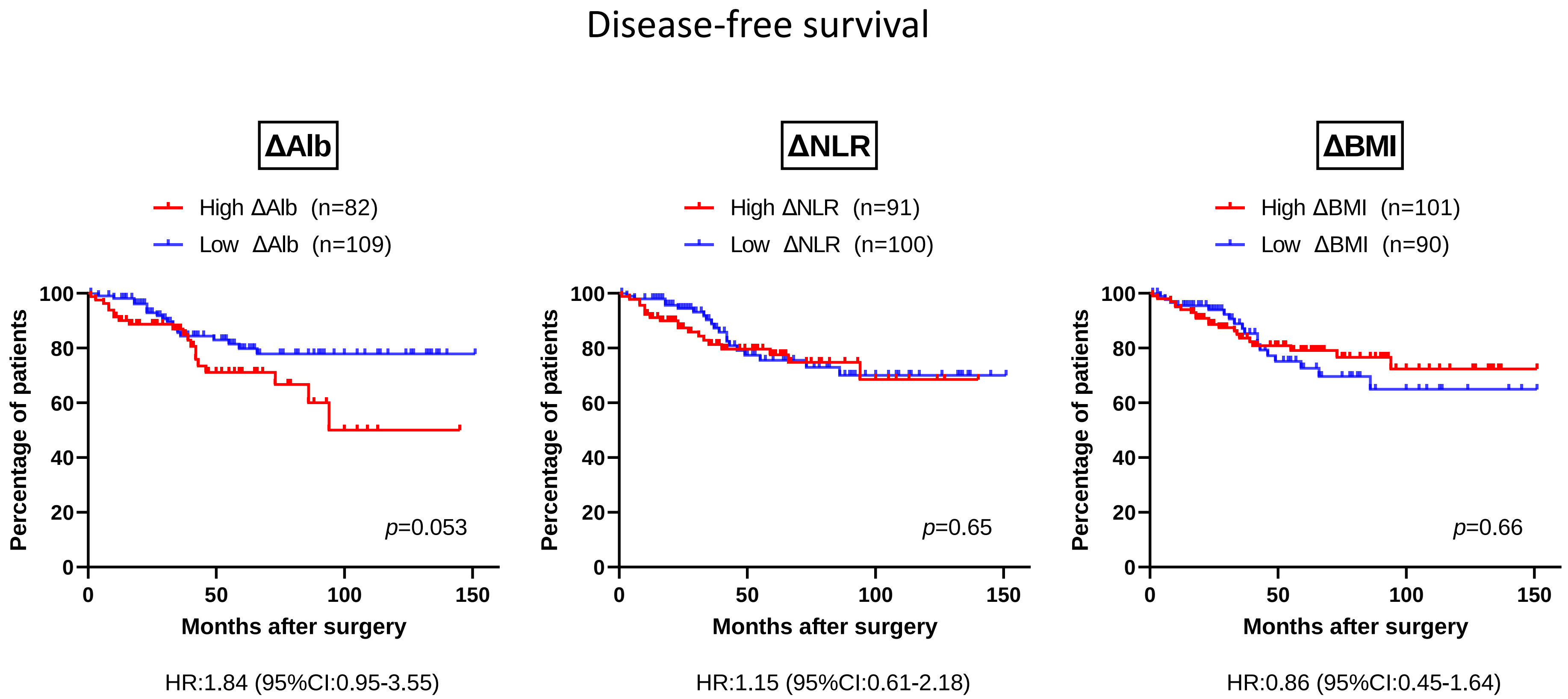
Disease-free survival
ΔAlb
High ΔAlb (n=82)
Low ΔAlb (n=109)
p=0.053
HR:1.84 (95%CI:0.95-3.55)
ΔNLR
High ΔNLR (n=91)
Low ΔNLR (n=100)
p=0.65
HR:1.15 (95%CI:0.61-2.18)
ΔBMI
High ΔBMI (n=101)
Low ΔBMI (n=90)
p=0.66
HR:0.86 (95%CI:0.45-1.64)
Percentage of patients
Months after surgery
0
20
40
60
80
100
50
150
