# Supplementary material for: Neoadjuvant chemotherapy-induced decrease of prognostic nutrition index predicts poor prognosis in patients with breast cancer
Source: BMC Cancer. 2020 Feb 27;20:160. doi: 10.1186/s12885-020-6647-4 (PMC7045374; doi:10.1186/s12885-020-6647-4)
Supplement: Supplementary file 9 — Additional file 9: Figure S7. Kaplan–Meier curves for disease-specific survival according to change in Alb, NLR, and BMI. Alb: Serum albumin level (g/dl), NLR: Neutrophil/lymphocyte ratio, BMI: Body mass index. [file 12885_2020_6647_MOESM9_ESM.pdf]

Disease-specific survival

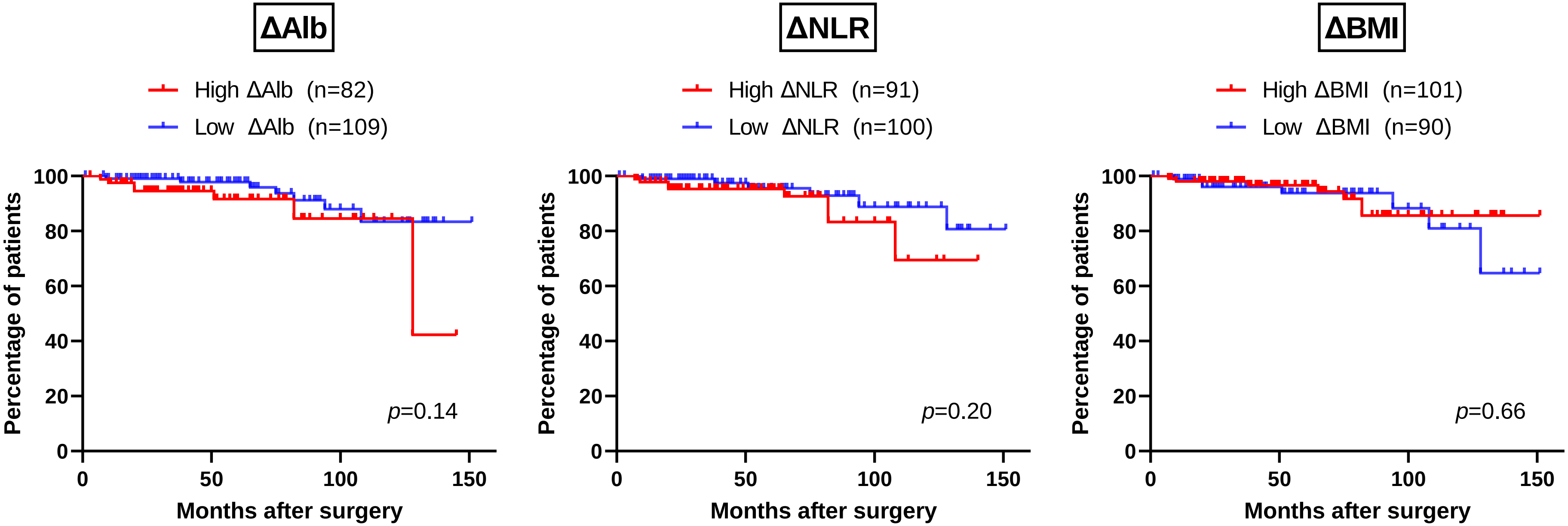


HR:2.09 (95%CI:0.68-6.43)

HR:1.92 (95%CI:0.66-5.58)

HR:0.79 (95%CI:0.27-2.28)
