# Supplementary material for: Neoadjuvant chemotherapy-induced decrease of prognostic nutrition index predicts poor prognosis in patients with breast cancer
Source: BMC Cancer. 2020 Feb 27;20:160. doi: 10.1186/s12885-020-6647-4 (PMC7045374; doi:10.1186/s12885-020-6647-4)
Supplement: Supplementary file 14 — Additional file 14: Figure S12. Kaplan–Meier curves for disease-free survival according to change of PNI by clinical stage and HG. PNI: Prognostic nutritional index, HG: Histological grade. [file 12885_2020_6647_MOESM14_ESM.pdf]

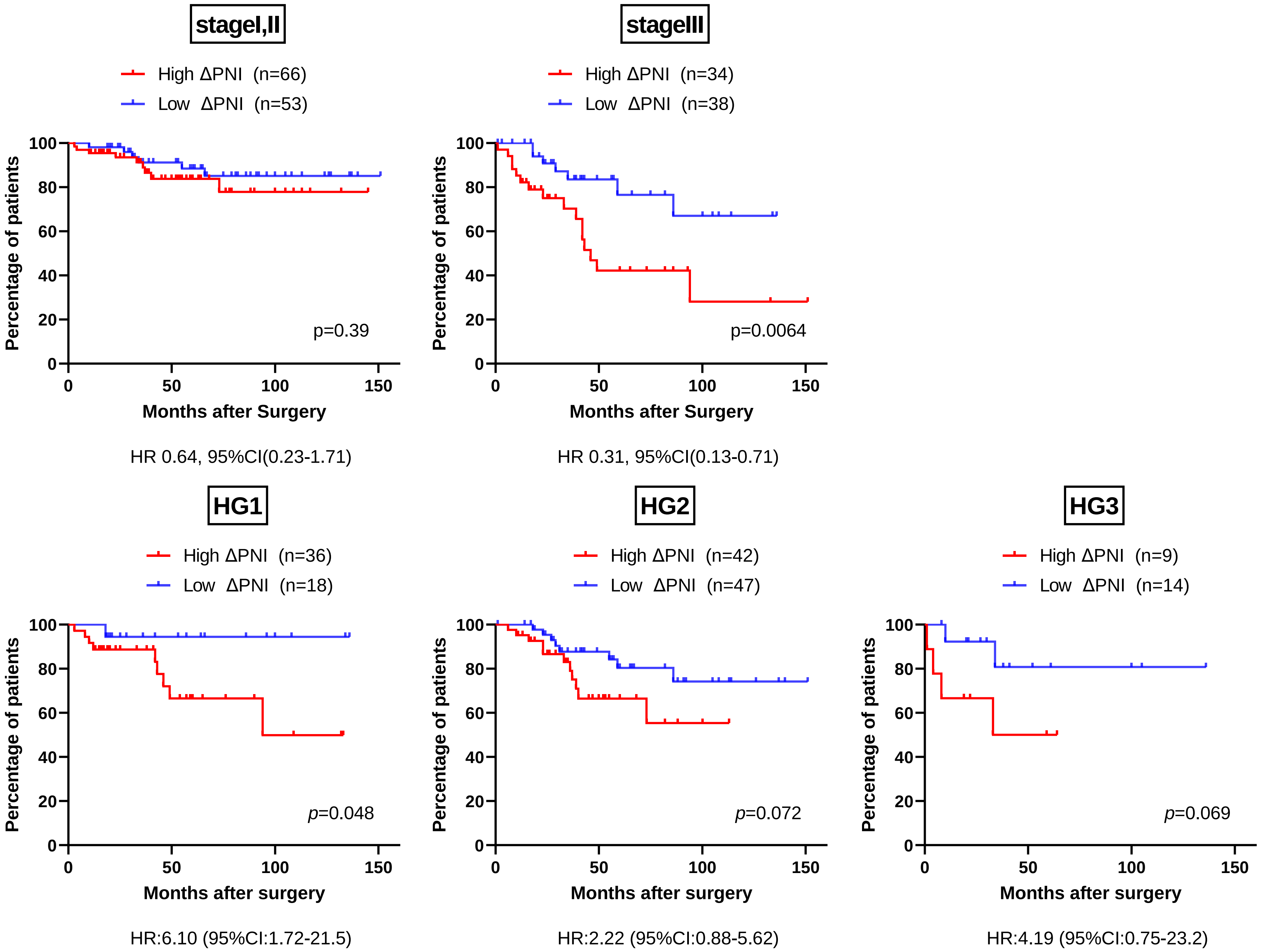
stageI,II
High ΔPNI (n=66)
Low ΔPNI (n=53)
Percentage of patients
Months after Surgery
p=0.39
HR 0.64, 95%CI(0.23-1.71)
stageIII
High ΔPNI (n=34)
Low ΔPNI (n=38)
Percentage of patients
Months after Surgery
p=0.0064
HR 0.31, 95%CI(0.13-0.71)
HG1
High ΔPNI (n=36)
Low ΔPNI (n=18)
Percentage of patients
Months after surgery
p=0.048
HR:6.10 (95%CI:1.72-21.5)
HG2
High ΔPNI (n=42)
Low ΔPNI (n=47)
Percentage of patients
Months after surgery
p=0.072
HR:2.22 (95%CI:0.88-5.62)
HG3
High ΔPNI (n=9)
Low ΔPNI (n=14)
Percentage of patients
Months after surgery
p=0.069
HR:4.19 (95%CI:0.75-23.2)
